# Preferences and reasons for clothing colors in women patients and survivors of breast cancer in Korea

**DOI:** 10.1371/journal.pone.0357499

**Published:** 2026-09-08

**Authors:** Seon-Hye Won, Yun-A. Kim, Sang-Yeon Suh, Ji Hyun Im, Erika Nakanishi, Tae Seon Lee, Yoonjoo Kim, Hee Jung Kim, David Hui, Beom Seok Kwak

**Affiliations:** 1 Department of Family Medicine, Dongguk University Ilsan Hospital, Goyang, Republic of Korea; 2 Department of Family Medicine, Daegu Catholic University School of Medicine, Daegu, Republic of Korea; 3 Department of Medicine, Dongguk University Medical School, Seoul, Republic of Korea; 4 Hospital School, Korea University Guro Hospital, Seoul, Republic of Korea; 5 Department of Palliative Nursing, Health Sciences, Tohoku University Graduate School of Medicine, Sendai, Japan; 6 Graduate School of Public Health, St. Luke’s International University, Tokyo, Japan; 7 Department of Neurosurgery, Severance Hospital & Yonsei University College of Medicine, Seoul, Republic of Korea; 8 Center for Medical Education, Graduate School of Medicine, Nagoya University, Nagoya, Japan; 9 Department of Nursing, College of Nursing, Dongguk University WISE, Gyeongju, Republic of Korea; 10 Kyungpook National University Hospital, Daegu, Republic of Korea; 11 Department of Palliative Care, Rehabilitation and Integrative Medicine, The University of Texas MD Anderson Cancer Center, Houston, Texas, United States of America; 12 Department of surgery, Dongguk University Ilsan Hospital, Goyang, Republic of Korea; The Chinese University of Hong Kong, HONG KONG

## Abstract

**Purpose:**

Clothing color can affect the psychological aspects in female patients and survivors of breast cancer; however, little is known regarding their preferences and reasons for choosing specific clothing color. This study aimed to explore the factors influencing their clothing color choices.

**Method:**

This qualitative study was conducted through face-to-face interviews. Fourteen adult female patients and survivors of breast cancer were recruited at a university hospital in Korea. An art therapist requested participants to indicate their usual choice of clothing colors and reasons. Participants were encouraged to talk about memories related to clothing colors in the past and present. Participants were asked if there were any changes in clothing colors after the diagnosis of breast cancer. Preferences and reasons regarding clothing colors were explored during one-hour interview. The interviews were audiotaped and analyzed using the Korean Computer Assisted Qualitative Data Analysis Software (CAQDAS), Blue Bird 2.0 (www.thebluebird.kr). The researchers crosschecked thematic coding results using the software.

**Results:**

All 14 patients and survivors had memorable experiences regarding their favorite clothing colors. Thematic coding identified four key themes: (1) social interactions and social image (39.5% of key sentences stated), (2) enhanced self-image and concealment of body shape (20.9%), (3) positive feelings (18.6%), and (4) changes in color preference after breast cancer diagnosis (14%).

**Conclusion:**

All female patients and survivors of breast cancer had preferences and good memories for clothing color. In most participants, these colors supported social interaction, self-image, and positive feelings. It is needed to investigate if intervention through clothing colors would support quality of life.

## Introduction

The incidence of breast cancer has been on the rise in Korea. It has been the most common cancer among Korean women. The number of newly diagnosed female breast cancer cases rose from approximately 17,214 in 2013–29,510 in 2023 [1]. Thus it showed nearly a 1.7 fold increase over the last decade [2]. Breast cancer belongs to a group of cancers with favorable prognosis, which is attributed to the advances in treatment options. In Korea, the 5-year survival rate of patients with breast cancer reached 93.6% for those diagnosed in 2017–2021 [1,2]. According to the Korean Breast Cancer Society registry, approximately two-thirds of patients underwent breast-conserving surgery while one-third received mastectomy in 2021 [1]. This is accompanied by an increasing number of breast cancer patients and survivors. These patients suffer from psychological distress during the diagnosis and treatment of cancer. Particularly, breast cancer is known to affect the overall quality of life (QoL) of patients more than any other type of cancer [3]. As breasts provide feminine symbolic meaning to women, the patients and survivors of breast cancer may experience emotional and social dysfunction after mastectomy. Alterations in the physical appearance may lead to a negative self-image and difficulties in relationships with partners and family members. Consequently, several patients with breast cancer reported distress due to their significant influence on body image, sexuality, and maternity [4]. Thus, it is not surprising that depression and anxiety are highly prevalent, accounting for approximately 50% of women with breast cancer [5]. Previous studies have found that isolation and low social support are factors associated with psychological distress [6–8]. Moreover, social support has been shown to improve QoL [9].

Various psychosocial interventions have been introduced to enhance the emotional well-being of women with cancer. For example, self-help groups and cognitive-behavioral approaches have been used to promote positive lifestyle changes. In this context, ‘Look Good Feel Better’ was developed as an extended public service to help patients with cosmetic education from volunteers [10]. In Korea, a controlled study reported positive effects of a cosmetics education program [11]. The investigators found that both distress and negative coping were reduced in the treatment group. Facial makeup is known to activate the brain through several sensations, such as sight, touch, and smell. Make-up and clothing are part of women’s daily lives, and both are related to self-image and social interactions. Especially, clothing plays a key role in expressing personal identity, and social status, while also facilitating social interaction by conveying one’s self-image and how they believe others perceive them [12]. Another common characteristic is color selection. Colors have been linked to mood, cognition, and behavior through their psychological functioning [13]. Environmental color, as well as changes in hair and skin color, have been associated with QoL in patients with cancer [14–16]. However, little is known regarding the preferences of women and their reasons for the clothing color choices before and after the diagnosis of cancer [17,18]. To the best of our knowledge, no studies have investigated future expectations regarding the clothing color in patients with breast cancer. To date, preceding literature has focused mainly on body image perception, self-esteem, and appearance-related interventions such as cosmetic or makeup programs [19,20]. In contrast, clothing color preference represents a daily behavioral expression of self-image that can shape social interactions, yet this aspect remains unexplored. Therefore, a qualitative study of the clothing colors of women with breast cancer is needed. The present study explored women’s real-life experiences of clothing color preference, focusing on how these choices related to self-identity, coping strategy and social feedback. We believe that it could be a baseline approach for potential interventions to enhance the psychological well-being of women with cancer in the near future.

This study aimed to investigate the preferences, reasons for preferences, change after morbidity of clothing colors from participants’ views. We planned to approach the perspectives of female patients and survivors of breast cancer at a university hospital through interview.

## Methods

This study employed a phenomenological approach to explore participants’ experiences through interviews. Two experts in palliative care research (SYS and DH) reviewed the literature and discussed the methodology used to approach clothing color. As little is known in this field, we chose a qualitative method to explore this theme using the patients’ own narratives. A total of 11 patients and 3 survivors of breast cancer were enrolled at a university hospital in the metropolitan area of Korea from July to September 2017. We included female patients or survivors of breast cancer with an interest in art therapy. Individuals who declined to participate in the art therapy sessions were excluded. A breast surgeon (BSK) introduced the study to eligible patients in both the ward and outpatient clinic. Only those who expressed interest in art therapy and provided written informed consent were included. The number of eligible patients who declined participation was not recorded. Participants were recruited consecutively after screening of interest in art therapy program, there was no rejection among those who showed interest in it. The research period was confined to which the art therapist was available. An art therapy session was conducted for the participants to facilitate interviews. A qualified art therapist (JHI) conducted an art therapy session. The art therapist conducted a one-hour interview during the session. The art therapy session was named “I am a flower.” The participants were asked about their favorite flowers. They expressed themselves as flowers using pastels, crayons, or colored clay, with the aid of an art therapist. The study participants were encouraged to talk about memories of their clothing colors in the past and present. The participants were asked if there were any changes in the selection of colors after being diagnosed with breast cancer. Specifically, the participants were encouraged to speak freely about preferences for clothing colors and reasons before their diagnosis, and to describe whether and how these had changed after diagnosis. Pre versus post-diagnosis preferences were compared through thematic coding of participants’ narratives. The details of the interview questions are shown in S1 File. All the interviews were audiotaped by the art therapist during the session. Audio files were transcribed using a web-based program (www.clovanote.com) [21]. The principal investigator (SYS) uploaded the transcripts into the Korean Computer-Assisted Qualitative Data Analysis Software (CAQDAS), Blue Bird 2.0 (www.thebluebird.kr) [22]. Three researchers (SHW,YAK and SYS) identified frequently repeated sentences across interviews. Above three major researchers chose repeated sentences independently, and compared each other’s key sentences together later. When disagreement arose, authors had a consensus meeting. They discussed about discrepancies and clarified key concepts till they reached consensus. Researchers put key sentences manually into Blue Bird 2.0. Then the software detected the recurrence of similar expressions across 14 transcripts of all participants. This process is technically automated function of the software; searching and counting frequencies in all transcripts. This allowed transparent and traceable coding results. Theme and subtheme classification was subsequently performed by the major researchers through interpretive analysis. A trained research nurse (YK) and research assistant (HJK) cross-checked the thematic analysis results. Researchers engaged in reflexive discussions to interpret the data. An experienced clinical psychologist (TSL) participated in discussing the influence of clothing color on psychological well-being. A nurse and researcher experienced in palliative care (EN) joined to discussing clinical insights and overall concepts. All researchers checked and discussed the thematic analysis process and results of themes and subthemes. S2 File provides the overview of the analysis process, and the thematic coding framework (S2 File, S1 Table). All the participants provided written informed consent. The art therapist explained to the participants about confidentiality measures and that all interviews would be audiotaped; these dialogues would be dealt with as anonymous data and would be accessible to authorized research members only. All recordings were transcribed and de-identified prior to analysis. Participants were assigned as sequential numbers (Participant 1 through Participant 14). We erased potentially identifiable personal information from the transcripts. Transcripts and coding materials were stored on a secure server which authorized personnel only have access. The institutional review board (IRB) of this study site approved the study (IRB number: DUIH 2017-01-060-004).

## Results

### Participants’ characteristics

A total of 14 participants aged between 49–65 years took part in this study (Table 1). Among them, 11 patients were undergoing active treatment for breast cancer, while 3 survivors were being monitored for the recurrence of breast cancer. The average interview duration was approximately 57 minutes. Most patients (7/14, 50.0%) had stage III breast cancer, and 92.9% of the patients were histologically confirmed to have invasive ductal carcinoma. Notably, 13 of 14 patients (92.9%) underwent surgery, 3 patients (21.4%) underwent total mastectomy, 5 patients (35.7%) underwent breast-conserving surgery, and 5 patients (35.7%) underwent modified radical mastectomy. In terms of the adjuvant therapy after surgery, 12 patients (85.7%) received chemotherapy, 10 patients (71.5%) received radiation therapy, and 8 patients (57.1%) received hormonal therapy.

**Table 1:** Clinical characteristics of the participants (n = 14).

Characteristics	n (%)
**Age** (years, mean ± SD)	55.2 ± 4.8
≤50 years	3 (21.4)
>50 years	11(78.6)
Surgery (Breast, Axilla)*	
Total Mastectomy	3 (21.4)
Breast conserving surgery	5 (35.7)
Modified radical mastectomy	5 (35.7)
Sentinel lymph node biopsy	6 (42.9)
Axillary lymph node dissection	2 (14.3)
Adjuvant therapy*	
Chemotherapy	12(85.7)
Radiation therapy	10(71.5)
Hormonal therapy	8 (57.1)
Histology*	
Invasive ductal carcinoma	13 (92.9)
Invasive lobular carcinoma	1 (7.1)
Paget’s disease	1 (7.1)
Stage**	
ⅡA	3 (21.4)
ⅡB	1 (7.1)
ⅢA	3 (21.4)
ⅢC	5 (35.7)
IV	1 (7.1)

Values are presented as numbers (percentage).

Age is presented as mean± standard deviation (SD).

*Duplicate entries possible.

**One participant was diagnosed of Paget’s disease.

### Key themes

We observed that the patients’ preferences for clothing colors reflected their memorable experiences in this qualitative study. The interview reminded participants of agreeable moments and surroundings related to the color of their clothes. Key themes were extracted from the participants’ own words using Blue Bird 2.0 software in Korean. A total of 43 important sentences were identified across all interviews, many of which overlapped conceptually. Researchers classified all codes into themes and subthemes using the software. The four main themes and associated subthemes are shown in Table 2.

**Table 2:** Themes and subthemes identified from the interviews (n = 14).

**1. Social interactions and social image**	1-1. Receiving compliments and drawing attention	13/43 (30.2)	11 (78.6)
	1-2. Aligning clothing with professional expectations	4/43 (9.3)	4 (28.6)
**2. Enhanced self-image and concealment of body shape**	2-1. Achieving a well-coordinated, comfortable style	4/43 (9.3)	3 (21.4)
	2-2. Concealing or minimizing perceived flaws in body shape	5/43 (11.6)	6 (42.9)
**3. Positive feelings**	—	8/43 (18.6)	6 (42.9)
**4. Changes in color preference after diagnosis**	—	6/43 (14.0)	5 (35.7)

*Percentages were calculated using the total number of the key sentences (n = 43) as the denominator.

**Participants, n indicates the number of participants contributing at least one or more key sentence to each theme/subtheme. Percentages used total participants (n = 14) as the denominator; categories were not mutually exclusive.

See section 2. Denominator and frequency convention in S2 File for more explanation.

## Theme 1: Social interactions and social image (39.5%, 17/43 key sentences)

Two subthemes were identified here: (1) social interactions with others, and (2) social image related to professional expectations.

### Subtheme 1−1. Social interactions with others: receiving compliments and drawing attention from others (30.2%, 13/43 key sentences)

*People compliment me a lot when I wear pink. I have pink dresses and I even have a matching pink umbrella. People like it. (Participant 1, 54-year-old, stage IIb breast cancer)*

*My husband often complimented me when I wore navy colored clothes. I have been wearing navy color ever since. (Participant 4, 59-year-old, stage IIIc breast cancer)*

*It’s a dark, neutral color, but since it’s a high-end piece, people keep saying it looks really stylish. (Participant 5, 65-year-old, stage IIIa breast cancer)*

*People complimented me when I wore bright color. They said things like “you look pretty” or “you look healthy.” (Participant 6, 49- year-old, stage Ⅲc breast cancer)*

*People said I look fabulous when wearing bright color. (Participant 7, 61-year-old, Paget’s disease)*

*I try to wear glittering clothes. I like the feeling of standing out and being noticed. I definitely get recognized when people notice me because of the color of my clothes. … such as red… (Participant 9, 57-year-old, stage IIIa breast cancer)*

*Everyone told me ‘Red makes you look fancy and bright, it suits you very well.” (Participant 12, 58-year-old, stage Ⅲc breast cancer)*

### Subtheme 1–2. Social image; aligning clothing with professional expectations (9.3%, 4/43 key sentences)

*Because I’m on stage almost every day, I prefer wearing bright colors rather than dark ones, as the brighter ones look better. (Participant 9, 57-year-old, stage Ⅲa breast cancer)*

*I run my own business, and I need to be shown slim and fit. I mostly buy black clothes to cover up. (Participant 10, 52-year-old, stage Ⅲa breast cancer)*

*I’ve been in the telecommunication business for years. I have to wear something bright to look attentive. (Participant 12, 58-year-old, stage Ⅲc breast cancer)*

*I often wear black and white because of my work in the wedding industry. (Participant 13, 50-year-old, stage IIIc breast cancer)*

## Theme 2: Enhanced self-image and concealment of body shape (20.9%, 9/43 key sentences)

This theme comprised of two subthemes: (1) achieving a well-coordinated and comfortable style, and (2) using clothing to minimize perceived flaws in body shape.

### Subtheme 2−1. Achieving a well-coordinated, comfortable style (9.3%, 4/43 key sentences)

*I think white makes people look lighter and cleaner. It especially suits me and has the same ‘looking-clean’ effect on myself. It also makes me stand out with whitish brightness. (Participant 1, 51-year-old, stage IIb breast cancer)*

*The navy colored dresses look neat and fit me well. (Participant 4, 59-year-old, stage Ⅲc breast cancer)*

*Sky blue suits me well, I feel comfortable and so fresh when I wear blue. (Participant 8, 57-year-old, Stage IIIc breast cancer)*

*Orange is bright color, it looks good on me, and I also like it. (Participant 11, 53-year-old, stage IIa breast cancer)*

### Subtheme 2−2. Using clothing to cover or minimize perceived flaws in body shape (11.6%, 5/43 key sentences)

*Even though I don’t have a specific preference, I choose colors that make me look slimmer and fit. (Participant 1, 54-year-old, stage Ⅱb breast cancer)*

*It seems I started wearing dark-colored clothing because I gained weight. (Participant 2, 49-year-old, stage Ⅳ breast cancer)*

*I often go for black, simply because I have a bigger frame and used to be on the chubby side.*

*(Participant 6, 49- year-old, stage Ⅲc breast cancer)*

## Theme 3: Positive feelings (18.6%, 8/43 key sentences)

This theme focused on participants’ emotional responses to clothing colors; how preferred colors enhanced self-confidence, positive outlook.

*Bright and vibrant colors give off confidence, I somehow feel confident wearing them. It just feel*s *like something good is going to happen. (Participant 1, 54-year-old, stage Ⅱb breast cancer)*

*Wearing brighter colors might improve my mood and potentially influence my personality to become more cheerful. It feels like wearing pretty colors make my mind feel more beautiful. (Participant 2, 49-year-old, stage Ⅳ breast cancer)*

*I feel lighter and uplifted when I try white or pastel-toned clothes. It makes my face look lighter, and my feelings and thoughts follow this brightness. (Participant 6, 49-year-old, stage IIIc breast cancer)*

*Red color gives off a dynamic impression on a person. I feel more active. (Participant 9, 57-year-old, stage Ⅲa breast cancer)*

*A vague expectation of something good arises just because I wear bright colors. (Participant 14, 58-year-old, stage Ⅱa breast cancer)*

## Theme 4: Changes in color preferences after breast cancer diagnosis (14%, 6/43 key sentences)

*From the moment I was diagnosed with breast cancer, I developed a preference for fancy floral patterns in chest of my clothes. I believed this might serve as an eye-catching motif to draw attention away from my diagnosis. (Participant 6, 49- year-old, stage Ⅲc breast cancer)*

*After learning about my breast cancer diagnosis, I tended to prefer darker colors in my clothing. (Participant 7, 61-year-old, Paget’s disease)*

*For me, there is a preconceived notion that my breast should be covered. But wearing something colorful and vibrant helps divert other people’s attention. (Participant 11, 53-year-old, stage IIa breast cancer)*

## Discussion

All female patients and survivors of breast cancer showed their own preferences and reasons for clothing color in this study. The most prominent theme (39.5%) was social interactions with others, such as favorable feedback and pleasant experiences. Enhanced self-image and concealment of body shape were second common. Subsequently, positive feelings appeared as approximately 19%. The last reason was changes after the diagnosis of breast cancer for clothing color preference. As this was a qualitative interview study, participants’ accounts were based on retrospective memories rather than contemporaneous observation. In our analysis, participants’ descriptions of clothing colors before diagnosis were compared with those after diagnosis to identify perceived changes in their preferences of the color.

1) Social interactions and social image: We observed that the participants retrieved enjoyable memories related to their clothing color, such as receiving compliments or pleasant social interactions with others. People not only consciously select a color of clothes to serve a purpose and to portray their self-image, but also to interact with others and mingle with their surroundings [23]. Among the 43 key sentences, 17 were related to positive comments from closely related people regarding their clothing color. For instance, the participants said: “*People compliment me a lot when I wear pink.”, “People said I look fabulous when wearing bright color”.* Those descriptions reflected happy memories of our participants about their relationships with surrounded people.
2) Enhanced self-image and concealment of body shape: Approximately 21% of the key sentences indicated that clothing color was chosen to achieve a self-satisfactory appearance as evidenced by participants’ remarks such as *“fit me well”* and *“that make me look slimmer and fit”.* Regardless of breast cancer diagnosis, participants tended to focus on their pre-diagnosis body shape. Previous studies have shown that higher body mass index and self-reported weight were linked to an increased preference for clothing aimed at hiding the body [24]. This aligns with our findings of participants tending to choose dark-colored clothing using their own words, such as *“I started wearing dark-colored clothing because I gained weight”.* Clothing can also reduce the gap between an individual’s actual and ideal self-image, leading to increased confidence and self-satisfaction [12].
3) Positive feelings: Of all key sentences, 19% pertained to themes associated with positive emotional experiences. Participants expressed their favorite colors foster feelings of positivity, self-confidence, and engagement with life. Those were referred as “*give off confidence*”, and “*something good is going to happen*”. Our findings are consistent to previous research suggesting that clothing serves as a medium for women to cultivate a healthy self-perception and enhance their self-esteem [25]. Self-esteem is closely linked to emotional well-being in female patients with breast cancer [26]. Therefore, selecting favorite clothing colors may help improve not only emotional well-being but also overall QoL.
4) Changes in color preferences after breast cancer diagnosis: After breast cancer diagnosis, the participants described “*eye-catching motif to draw attention away from”* and “*divert other people’s attention*” in their talk, as reasons for clothing colors and patterns. Our participants chose specific colors and patterns to divert attention away from the breasts, which can be compared with using concealment devices such as wigs or scarves to disguise altered body image [27,28]. Most breast cancer patients and survivors experience negative changes in appearance, such as body image, skin alteration, and hair loss [29]. In particular, breast surgery causes a significant loss of body image in women [30]. These changes can lead to decreased self-image and self-esteem [31,32]. However, this pattern was not universal among participants. In our sample, seven of fourteen participants reported no meaningful change in their clothing color preferences after diagnosis, two were unsure because they had not yet resumed usual clothing purchases after recent surgery, and five described a clear shift toward darker tones, vivid floral patterns, or attention-drawing colors. These mixed responses suggest that clothing color preference may reflect both stable personal style and adaptive coping behavior following breast surgery.

Past studies have proved that social support improved the QoL of patients with breast cancer by promoting their self-esteem [33]. Especially, those patients are known to have a feeling of social withdrawal and lower QoL after diagnosis [34]. Thus, female patients with breast cancer could try using their favorite colors in diverse clothes to shape a better body image. As body image is a known predictor of psychological functioning in women with breast cancer [19], encouraging their pleasant experiences of favorite clothing color may lead to a better body image [35,36]. Until now, a few previous studies have reported significant effects of color selection of patients in various disease groups through art therapy [37–39]. Previously, investigators reported a positive effect of cosmetic education on women with breast cancer [40]. The investigators concluded that cosmetics uplifted the patients’ moods by the enhancement of their self-image. We agree that color could be a good therapeutic tool for such cognitive intervention. Because participants’ statements about their favorite clothing colors brought back a positive spirit related to social support and enhanced self-image. Therefore, we presume that further intervention through clothing color can boost female patients’ self-confidence of “looking good” and “feeling better”. As color is an essential part of everyone’s life and reflects personal characteristics and social interaction, reaching out to female patients with a medium of clothing color can be a helpful approach.

Cultural context may be background of these preferences. Korean color symbolism is rooted in the traditional Obangsaek, which means five cardinal colors (white, black, blue, red, yellow), with deep ties to Confucian and Yin–Yang traditions [41]. Korean national people had historically been identified as the “white-clad people”, the white color has been known for the symbol of purity in Korea [42]. In the Korean national flag (Taegukgi), red and blue directly express the cosmological principle of Yin-Yang. Red represents Yang, the active and generative cosmic force, while blue represents Yin, the receptive and complementary force [43]. Thus Koreans may regard red and blue are associated with vitality and positive energy. Given this context, clothing color may be used as an active effort to sustain social identity and regulate emotion [44–46]. Also it can be related to coping strategies when the body has undergone change [47]. However, color symbolism varies substantially across cultures, it warrants further investigations whether the findings are generalizable in other populations [48,49].

We suggest that clinicians may incorporate questions about clothing colors as one of coping strategies for women after diagnosis of breast cancer. Nurses, clinical psychologists and art therapists can encourage patients to express positive experiences related to clothing color: then patients would exchange supportive feedback. Patients can share their experiences about clothing color in peer support group with the same disease.

### Limitations

This study has some limitations. First, this study was conducted in South Korea. Thus, women’s preferences and expectations of clothing color may differ by country. Second, our sample size was relatively small and included convenience samples from a single institution. The number of eligible patients who declined participation was not recorded, which may further limit the transferability of our findings to broader populations. Therefore, our results should be regarded as hypothesis generating. Further studies with larger populations of various backgrounds and experiences based on purposive sampling are necessary. Third, our participants were at different stages of breast cancer treatment, ranging from two months to six years after diagnosis. Further qualitative studies should be homogeneous in terms of the period after diagnosis. Additionally, the inclusion criterion of interest in art therapy may introduce selection bias. Participants who volunteered may be more interested in color, thus our findings might be a bit different for all women with breast cancer. The study relied on retrospective self-report, thus their memories may be subject to recall bias. Memories of pre-diagnosis states might have been affected by experiences of diagnosis and treatment. We acknowledge that other factors may contribute to changes in color preference. Aging and adverse events from treatment -such as hair loss, skin discoloration- can all influence choice of clothing color. We hope future quantitative work could adjust those confounders.

Fourth, participant member-checking could not be implemented due to time constraints. Finally, other confounding factors may influence feelings and social interaction of patients with breast cancer. Those may include physical symptoms, psychiatric disorders, adverse events from cancer treatment and financial distress, etc [50]. Unfortunately, detailed information on these factors was not available to our participants. Moreover, as noted in the Discussion, only a subset of participants described changes in clothing color preferences after diagnosis, while others maintained stable choices. Therefore, color preference may partly reflect individual style rather than cancer-related adjustment. Future study should define and consider these factors for better understanding.

## Conclusion

This study showed that all female patients and survivors of breast cancer had preferences and reasons for clothing color. Evidently, clothing color is related to an individual’s feelings through their self-image and social interactions. And some participants used clothing color as an adaptive measure for breast cancer. Therefore, our findings suggest that clothing color can serve as a simple psychosocial approach to support positive QoL and coping strategies in women with cancer. Future studies should examine whether color-based interventions can enhance psychological well-being of women patients with cancer.

## Supporting information

S1 File Semi-structured Interview Guide. (DOCX)

S2 File Thematic Coding Framework. (DOCX)

S1 Table Theme and Subtheme Definitions with Coding Rules. (DOCX)
