# Supplementary material for: Preferences and reasons for clothing colors in women patients and survivors of breast cancer in Korea
Source: PLoS One. 2026 Sep 8;21(9):e0357499. doi: 10.1371/journal.pone.0357499 (PMC13552724; doi:10.1371/journal.pone.0357499)
Supplement: Supplementary file 1 [file pone.0357499.s001.docx]

**Supplementary File 1. Semi-structured Interview Guide**

We would like to ask about your usual thoughts and experiences about clothing colors. Please talk freely and express whatever comes to mind.

**1.** Throughout your life, what colors of clothing have you mainly worn? What were the reasons for your choices?

**2.** Throughout your life, which clothing colors do you believe suits you best? Please explain the reason for your answer.

**3.** Since your breast cancer diagnosis and during your subsequent treatment, have you noticed any changes in your preference or selection of clothing colors? If so, what factors influenced this change?

**4**. Have you ever experienced a change in your mood based on the color of the clothing you wore, or have you received compliments from those around you regarding your choice of attire? If yes, please describe the experience in detail.
