# Supplementary material for: Preferences and reasons for clothing colors in women patients and survivors of breast cancer in Korea
Source: PLoS One. 2026 Sep 8;21(9):e0357499. doi: 10.1371/journal.pone.0357499 (PMC13552724; doi:10.1371/journal.pone.0357499)
Supplement: Supplementary file 2 [file pone.0357499.s002.docx]

# Supplementary File 2. Thematic Coding Framework

## 1. Overview of the analysis procedure

Interviews were transcribed using a web-based program (ClovaNote: NAVER Corporation; <https://clovanote.naver.com>). The principal investigator (SYS) and the art therapist (JHI) verified those by comparing with the original recordings. Verified transcripts were uploaded into the Korean Computer-Assisted Qualitative Data Analysis Software (CAQDAS) Blue Bird 2.0 (Academy Press Coporation; www.thebluebird.kr), which tracked the recurrence of similar expressions across transcripts.

Three major researchers (SHW, YAK, SYS) independently identified repeated expressions across all interview transcripts. A total of 43 key sentences were identified across the 14 interviews. Then researchers classified key sentences into thematic clusters by semantic similarity and frequency. Thematic classification was researcher-driven and inductive; the software supported, but it did not replace human interpretation or judgment. They explained their interpretation for themes and subthemes to each other, while they recognized their own position and environment might influence the interpretation (reflexive discussion). Discrepancies were resolved through consensus meeting by major researchers till reaching consensus.. All authors double-checked and provided their expertise about the thematic analysis process and results of themes and subthemes.

Blue Bird 2.0 functioned as a data-management and frequency-tracking tool rather than an automated classifier. After the verified transcripts were uploaded, the major authors entered the key sentences manually; the software then detected the recurrence of similar expressions across all 14 transcripts, enabling systematic frequency comparison.

In this procedure, validity lies on the authors’ thematic coding, cross-checking, and consensus while the reliability of searching similarities and counting frequency is supported by the software’s automated tracking function. This software provided an auditable link between each key sentence and its source transcript to keep transparency. Blue Bird 2.0 is mostly used in the social science field in Korea and has supported a number of published studies.

## 2. Denominator and frequency convention

It is noteworthy that all reported frequencies and percentages refer to the proportion of the 43 coded key sentences attributed to each theme or subtheme (not the proportion based on the 14 participants). A separate “Participants, n” count indicates how many distinct participants contributed at least one key sentence to each theme or subtheme. Because a single participant could contribute several key sentences but single sentence could be assigned to one theme only. Thus we reported two counts in separate columns (Table 2).

## 3. Selected References of Qualitative Studies Using Blue Bird 2.0

1. Oh YB. Exploring the Meaning of Project-Based Learning through Project Class Cases. Korean Education. 2017.

2. Baek MW, Ha MJ. Teachers’ Dual Perceptions of Students from Multicultural Backgrounds. Journal of Multicultural Education Studies. 2016.

3. Kim YK. Voices of Three Arts Educators: Experiences, Perspectives, and Visions of Arts Education in Schools. Journal of Anthropology of Education. 2017.

4. Lee HC. A Narrative Inquiry into Youth Retreats in Korean Churches. Christian Education Journal. 2016.

5. Ahn SH. The Novice Art Therapists' Difficulties and Required Competencies: A Focus Group Interview. 2018.

6. Seok MJ, Jung MJ. A Van Manen Hermeneutic Phenomenological Analysis of the Home Escape Process among Women Experiencing Domestic Violence. Asia-Pacific Journal of Convergent Research Interchange. 2023.
