# Supplementary material for: Preferences and reasons for clothing colors in women patients and survivors of breast cancer in Korea
Source: PLoS One. 2026 Sep 8;21(9):e0357499. doi: 10.1371/journal.pone.0357499 (PMC13552724; doi:10.1371/journal.pone.0357499)
Supplement: Supplementary file 3 [file pone.0357499.s003.docx]

# Supplementary 2 Table

## S2 Table. Theme and subtheme definitions with coding rules

| **Theme / Subtheme** | **Definition** | **Coding rule** |
| --- | --- | --- |
| **1. Social interactions and social image** | Key sentences in which clothing color is described in relation to other people — feedback, attention, or alignment with a social or occupational role. | Include sentences related to others’ reactions or social/professional context of color choice. Exclude purely internal feelings (→ Theme 3) and statements about body concealment without a social referent (→Subtheme 2-2). |
| **1-1. Receiving compliments and drawing attention** | Color choices linked to compliments, praise, or being noticed by family, friends, or acquaintances. | Include explicit reports of compliments or visibility tied to a color. Exclude compliments attributed to style/fit rather than color. |
| **1-2. Aligning clothing with professional expectations** | Color choices according to occupational role, workplace norms, or social expectations for the job. | Include sentences tying color to a job, business, or stage/performance context. Exclude general social compliments (→Subtheme 1-1). |
| **2. Enhanced self-image and concealment of body shape** | Key sentences in which color serves the participant’s own appearance goals — looking coordinated and comfortable, or concealing perceived flaws of body. | Include self-referential appearance statements. Exclude colors chosen mainly for others’ reactions (→Theme 1). |
| **2-1. Achieving a well-coordinated, comfortable style** | Color chosen because it suits the participant, looks neat/clean, or feels comfortable. | Include “suits me / looks neat / fits well” statements. Exclude concealment of a specific flaw (→ Subtheme 2-2). |
| **2-2. Concealing or minimizing perceived flaws in body shape** | Color (often dark) chosen to make the body look slimmer or to hide a body-shape concern. | Include statements about slimming, covering weight gain. Exclude post-diagnosis concealment framed as an explicit change (→ Theme 4). |
| **3. Positive feelings** | Key sentences describing internal emotional responses drawn by preferred colors — confidence, positivity, uplift, or anticipation. | Include personal emotion/affect statements (“I feel confident / uplifted”). Exclude functional or socially oriented statements (→ Subtheme 1–2). |
| **4. Changes in color preference after diagnosis** | Key sentences in which the participant explicitly reports that their clothing-color preference changed after the breast cancer diagnosis and/or treatments. | Include only explicitly reported post-diagnosis shifts. Exclude “no change,” age-related and uncertainty due to not yet resuming clothing purchases. |
